# Pharmacological Administration of the Isoflavone Daidzein Enhances Cell Proliferation and Reduces High Fat Diet-Induced Apoptosis and Gliosis in the Rat Hippocampus

**DOI:** 10.1371/journal.pone.0064750

**Published:** 2013-05-31

**Authors:** Patricia Rivera, Margarita Pérez-Martín, Francisco J. Pavón, Antonia Serrano, Ana Crespillo, Manuel Cifuentes, María-Dolores López-Ávalos, Jesús M. Grondona, Margarita Vida, Pedro Fernández-Llebrez, Fernando Rodríguez de Fonseca, Juan Suárez

**Affiliations:** 1 Laboratorio de Medicina Regenerativa (UGC Salud Mental), Instituto de Investigación Biomédica (IBIMA), Complejo Hospitalario de Málaga (Hospital Carlos Haya), Pabellón de Gobierno, Málaga, Spain; 2 CIBER OBN, Instituto de Salud Carlos III, Ministerio de Ciencia e Innovación, Madrid, Spain; 3 Departamento de Biología Celular, Genética y Fisiología, Facultad de Ciencias, Universidad de Málaga, Málaga, Spain; 4 CIBER BBN, Instituto de Salud Carlos III, Ministerio de Ciencia e Innovación, Madrid, Spain; Hosptial Infantil Universitario Niño Jesús, CIBEROBN, Spain

## Abstract

Soy extracts have been claimed to be neuroprotective against brain insults, an effect related to the estrogenic properties of isoflavones. However, the effects of individual isoflavones on obesity-induced disruption of adult neurogenesis have not yet been analyzed. In the present study we explore the effects of pharmacological administration of daidzein, a main soy isoflavone, in cell proliferation, cell apoptosis and gliosis in the adult hippocampus of animals exposed to a very high-fat diet. Rats made obese after 12-week exposure to a standard or high-fat (HFD, 60%) diets were treated with daidzein (50 mg kg^−1^) for 13 days. Then, plasma levels of metabolites and metabolic hormones, cell proliferation in the subgranular zone of the dentate gyrus (SGZ), and immunohistochemical markers of hippocampal cell apoptosis (caspase-3), gliosis (GFAP and Iba-1), food reward factor FosB and estrogen receptor alpha (ERα) were analyzed. Treatment with daidzein reduced food/caloric intake and body weight gain in obese rats. This was associated with glucose tolerance, low levels of HDL-cholesterol, insulin, adiponectin and testosterone, and high levels of leptin and 17β-estradiol. Daidzein increased the number of phospho-histone H3 and 5-bromo-2-deoxyuridine (BrdU)-ir cells detected in the SGZ of standard diet and HFD-fed rats. Daidzein reversed the HFD-associated enhanced immunohistochemical expression of caspase-3, FosB, GFAP, Iba-1 and ERα in the hippocampus, being more prominent in the dentate gyrus. These results suggest that pharmacological treatment with isoflavones regulates metabolic alterations associated with enhancement of cell proliferation and reduction of apoptosis and gliosis in response to high-fat diet.

## Introduction

Obesity is a disease in which a positive energy imbalance results in excessive body fat accumulation, leading to reduced life expectancy and/or increased metabolic disorders such as hyperinsulinemia, insulin resistance and type II diabetes. Both obesity and saturated fats were just described to be factors determining direct injury in the brain [1], [2]. The discovery of antiobesity drugs capable of harmonizing central and peripheral networks controlling energy expenditure is therefore becoming an urgent need. While the prevalence of obesity is low in Asian countries, its frequency is raising in the Western world; such observation has turned the interest in Asian diets, which highly consist of soy and soy-based products [3]. The observed health benefits linked to soy consumption have been associated to its content in isoflavones, natural phytoestrogens that are structurally similar to estrogen (17β-estradiol or E2) and whose pharmacological properties as antiobesity agents are emerging [4].

Besides to the well-known reproductive role of gonadal hormones, estrogen also plays a critical role in brain development as well as in maintaining normal brain function in adulthood [5], [6]. Thus, it is noteworthy the neuroprotective role attributed to estrogens in a variety of brain injury models [7], [8]. Additionally, it has recently been established that gonadal hormones also influence adult neurogenesis. For instance, Tanapat et al. [9] found that female rats during proestrus had significantly more immature neurons in the subgranular zone (SGZ) of dentate gyrus (DG) than male rats did, suggesting that high levels of estrogen may have an influence on adult neurogenesis. Since then, emerging results indicate that estrogens influence on both cell proliferation and cell survival in DG of adult animals [10] or after stroke [11]. Many factors can influence the levels of adult neurogenesis. For instance, dietary modulation or physical exercise may influence cell proliferation in the hippocampus [12], [13]. Moreover, Rivera et al. [14] described a significant modulation of adult hippocampal, subventricular and hypothalamic neurogenesis associated to reduction of caloric intake and body weight gain after a 14-day treatment with the cannabinoid inverse agonist AM251 in rats fed a very high-fat diet (HFD).

In this context, physiologically attainable doses of isoflavones, behaving as phytoestrogens, can mimic some of the neuroprotective effects observed in 17β-estrogen [15] while promoting beneficial metabolic effects and reduction of body weight gain [4]. For example, daidzein, a major soy isoflavone, can activate estrogen receptors (ERs) and mimic the effects of the hormone in a lower scale [16]. Isoflavones have been shown to 1) protect primary hippocampal neurons from oxidative stresses induced by glutamate or β-amyloid [17], 2) attenuate the neurotoxic effect of D-galactose in mouse brain by decreasing caspase-3 expression in hippocampus and cortex [18] and 3) reduce astrogliosis induced by exogenous administration of β-amyloid protein [19].

Despite the metabolic action of isoflavones on energy balance and its influence on neuronal proliferation, there is still a general lack of knowledge regarding the regulatory role of phytoestrogens in adult neurogenesis in obese animals. In the present work we analyzed the potential effects of the pharmacological treatment with the isoflavone daidzein at a dose of 50 mg kg^−1^ for 13 days on cell proliferation in the SGZ of DG, cell apoptosis and gliosis in the hippocampus of rats fed standard (STD) and high-fat (HFD) diets for 12 weeks. The dose was selected on the basis of a previous study of this isoflavone on a feeding test [4].

## Materials and Methods

### Ethics Statement

The protocols for animal care and use were approved by the Ethic and Research Committee at the Hospital Carlos Haya and Universidad de Málaga. All experimental animal procedures were carried out in strict accordance with the European Communities directive 86/609/ECC (24 November 1986) and Spanish legislation (BOE 252/34367-91, 2005) regulating animal research. All efforts were made to minimize animal suffering and to reduce the number of animals used.

### Animals

Male Wistar rats (approximately 250 g, 10–12 weeks old; Charles Rivers, Barcelona, Spain) were housed individually in cages maintained in standard conditions (Servicio de Estabulario, Facultad de Medicina, Universidad de Málaga) at 20±2°C room temperature, 40±5% relative humidity and a 12-h light/dark cycle with dawn and dusk effect.

### Food/caloric Intake and Body Weight

Rats (*n* = 16 per group) were fed *ad libitum* for 12 weeks with two different types of diets: a very high-fat diet (HFD, 60% fat diet; D12492, Brogaarden, Gentofke, Denmark) and a standard diet (STD, 10% fat diet-D1245B). The accumulated food/caloric intake and the body weight gain were measured every 2–3 days for 12 weeks (Fig. S1).

### Subchronic Daidzein Treatment

After 10 weeks, half of each group of diet-fed rats (*n* = 8) received a daily intraperitoneal (i.p.) injection (09∶00 a.m.) of, either 50 mg kg^−1^ daidzein (7[hyphen]β-glucoside; LC Laboratories, Worburn, USA) dissolved in 10% Tocrisolve (Tocris, Bristol, United Kingdom) and saline, or vehicle (1 ml kg^−1^ of 10% Tocrisolve in saline) over 13 days, while the diets remained unchanged (Fig. S1). The dose was selected on the basis of the acute effects of this isoflavone on a feeding test [4]. Cumulative food/caloric intake and body weight gain were measured every day over the 13 days of treatment. Thus, the following four experimental groups were generated: STD-vehicle, STD-daidzein, HFD-vehicle and HFD-daidzein.

### Glucose Tolerance Test

At 30 minutes before starting the glucose tolerance test (GTT), 50 mg kg^−1^ daidzein or vehicle (1 ml kg^−1^ of 10% Tocrisolve in saline) was administered (i.p.) in 12-hour fasted rats fed STD and HFD. Twenty-five minutes later, tail blood samples were subsequently collected at basal level (0 minutes) and 5, 10, 15, 30, 60 and 120 minutes after a glucose overload (i.p.) at a dose of 2 g/kg body weight. Glucose levels were determined using a standard glucose oxidase method.

### BrdU Administration

5′-bromo-2′-deoxyuridine (BrdU, cat. no. B5002, Sigma, St. Louis, MO, USA) was dissolved at 15 mg ml^−1^ in 0.9% saline solution, and administrated (i.p.) at 50 mg kg^−1^ body weight twice per day at 10-hours intervals (08∶00, 18∶00 h), for 3 consecutive days (Fig. S1). The animals were killed 12 hours after the last injection of BrdU [20].

### Sample Collection

Previous to sacrifice, animals were anaesthetized (sodium pentobarbital, 50 mg kg^−1^ body weight, i.p.) two hours after the last dose of treatment in a room separate from the other experimental animals. Blood samples were briefly collected from the orbital cavity into tubes containing EDTA-2Na (1 mg/ml blood) and centrifuged (1600 *g* for 10 min, 4°C), and all plasma samples were frozen at −80°C for biochemical and hormonal analysis. Animals were then transcardially perfused with 4% paraformadehyde in 0.1 M phosphate buffer (PB) and brains were dissected and kept in the same fixative solution overnight at 4°C. Brains were then cut into 30-µm-thick coronal sections, divided in five parallel series, using a vibratome (Leica VT1000S). Sections were stored at 4°C in PB with 0.002% (w/v) sodium azide until further used for immunostaining.

### Biochemical and Hormonal Analysis

Plasma metabolites and hormones were measured using commercial kits according to the manufacturer’s instructions. Glucose, triglycerides, total cholesterol and high-density lipoprotein (HDL)-cholesterol were analyzed in a Hitachi 737 Automatic Analyzer (Hitachi Ltd., Tokyo, Japan) in the Hematology Service at the Hospital Carlos Haya (Málaga, Spain). Plasma insulin, adiponectin and leptin levels were determined with three different enzyme-linked immunosorbent assay (ELISA) kits from Mercodia AB (Uppsala, Sweden), B-Bridge International, Inc. (Mountain View, CA, USA) and BioVendor (Modrice, Czech Republic), respectively. To perform the ELISA protocols in rat samples, we used 25, 100 and 100 µl of plasma to determine the insulin, adiponectin and leptin concentrations, respectively. Plasma testosterone and 17β-estradiol levels were determined using an Immulite®2000 immunoassay system (Siemens, Tarrytown, NY, USA). We used 200 µl of rat plasma to perform the immunoassays. In all cases, a calibration curve and an internal control were included in each assay. Cross-reaction with daidzein was not detected.

### General Procedures for Immunohistochemistry

For the analysis of phospho-histone H3 and BrdU immunoreactivity in the SGZ of DG, and the immunohistochemical expression of total caspase-3, cleaved caspase 3, FosB, GFAP, Iba1 and ERα in the whole hippocampus, free-floating 30-µm-thick coronal sections from −2.12 to −4.16 mm Bregma level [21] were selected from one of eight parallel series obtained from each brain of the four experimental groups. Methodology and antibodies used were extensively described in Methodology S1.

### BrdU Immunohistochemistry

One immunostaining batch containing 8 sections per animal (*n* = 8, four groups) was stained simultaneously to avoid variations in the intensity of staining due to the procedures. Sections were incubated overnight in mouse anti-BrdU (1∶2000, Hybridoma Bank, Iowa City, IA, USA; ref. G3G4) at 4°C [14], [20]. The following day, sections were washed three times with PBS, incubated in biotinylated goat anti-mouse IgG (H+L) (1∶1000, Pierce, Rockford, IL, USA; cat. no. 31800) for 90 min, washed again in PBS, and incubated in avidin-biotin peroxidase complex (Pierce) diluted 1∶250 in PBT in darkness at room temperature for 45 min. Finally, immunolabelling was revealed with 0.05% diaminobenzidine (DAB; Sigma), 0.05% nickel ammonium sulfate and 0.03% H_2_O_2_ in PBS.

### Double Immunofluorescence

The following antibodies were used: rat anti-BrdU (1∶2000; Accurate Chemical & Scientific, Westbury, NY, USA) and mouse anti-β3 tubulin (1∶5000; Promega, Madison, WI, USA). Sections were incubated overnight at 4°C with a cocktail of the primary antibodies. The rat anti-BrdU was detected with goat anti-rat IgG labelled with Alexa 488 (1∶1000; Molecular Probes, Invitrogen, Paisley, UK). The antibody β3-tubulin was detected with donkey anti-mouse IgG Alexa 594 (1∶1000; Molecular Probes). The sections labeled by double immunofluorescence were visualized with a confocal microscope (Leica TCS NT; Leica Microsystems).

### Phospho-H3, caspase-3, FosB, GFAP, Iba 1 and ERα Immunohistochemistry

One immunostaining batch containing 5 sections per animals (*n* = 8, four groups) was stained simultaneously to avoid variations in the intensity of staining due to the procedures. Sections were incubated overnight in diluted primary antibody at 4°C: rabbit anti-phospho-histone H3 (Ser10) (2 µg/ml, Upstate, Lake Placid, NY, USA; cat. no. 06-570), rabbit anti-caspase-3 (1∶200, Cell Signaling Technology, Danvers, MA, USA; cat. no. 9662), rabbit anti-cleaved caspase-3 (1∶500, Cell Signaling; cat. no. 9661), rabbit anti-FosB (1∶2500, Santa Cruz Biotechnology, Santa Cruz, CA, USA; cat. no. sc-48), mouse anti-glial fibrillaric acidic protein (GFAP) (1∶500, Sigma; cat. no. G3893), rabbit anti-Iba-1 (1∶1000, Wako, Osaka, Japan; cat. no. 019-19741) and rabbit anti-estrogen receptor alpha (ERα) (1∶200, Santa Cruz Biotechnology; cat. no. sc-7207). The following day sections were incubated in the respective secondary antibody for 1 hour: biotinylated goat anti-mouse IgG (1∶500, Sigma; cat. no. B7264) and biotinylated donkey anti-rabbit IgG (1∶500, Amersham, Little Chalfont, England; cat. no. RPN 1004). Then sections were washed in PBS and incubated in ExtrAvidin peroxidase (Sigma, St. Louis, MO) diluted 1∶2000 in darkness at room temperature for 1 hour. Finally, immunolabelling was revealed with 0.05% diaminobenzidine (DAB; Sigma), 0.05% nickel ammonium sulfate and 0.03% H_2_O_2_ in PBS.

### Quantification of Phospho-H3, Caspase-3, FosB, GFAP and Iba1-immunoreactive Cells

Phospho-H3, caspase-3, FosB, GFAP, Iba1-immunoreactive (-ir) nuclei or cells that came into focus were manually counted using a standard optical microscope with the 40× objective (Nikon Instruments Europe B.V., Amstelveen, The Netherlands) coupled to the NIS-Elements Imaging Software 3.00 (Nikon). Phospho-H3 and BrdU-ir nuclei in the SGZ of dentate gyrus, and caspase-3, FosB, GFAP and Iba1-ir cells in the three hippocampal areas (DG, CA3 and CA1) were counted from −2.12 to −4.16 mm Bregma levels for each animal (*n* = 8). Nuclei and cell counts were performed in a single cerebral hemisphere in all cases. For GFAP immunostaining, a representative counting frame (40×) was evaluated in each area (DG, CA1 and CA3) and section analyzed. GFAP-ir cells located in the uppermost side that came into focus while moving down through the thickness of the section were counted. GFAP-positive fibers without a visible body cell were ignored. Overall, quantification was expressed as the total number of BrdU-ir cells or the average number of caspase-3, FosB, GFAP and Iba1-ir cells per area (mm^2^) for each experimental group.

### Quantification of GFAP and ERα Immunoreactivity

Digital high-resolution microphotographs of the hippocampus were taken with a 10× objective under the same conditions of light and brightness/contrast with an Olympus BX41 microscope equipped with an Olympus DP70 digital camera. Densitometric quantification of the immunoreactivity of representative areas was determined using the analysis software ImageJ 1.38X (NIH, USA). On each tissue section, we focused on CA1 and CA3 areas of Ammom’s horn and DG. For both CA areas, we considered the following layers: stratum oriens (SO), stratum pyramidale (SP), stratum radiatum (SR), stratum lucidum (SL) and stratum lacunosum-moleculare (SL-M). For DG, we considered following layers; the molecular layer (ml), the granular cell layer (gcl) and the polymorphic cell layer (pcl).

### Statistical Analysis

Data are represented as mean ± s.e.m. from at least six animals. Kolmogorov-Smirnov normality tests indicated that all data followed a Gaussian distribution (*P*>0.1), so we selected a parametric statistical test. Statistical analysis was performed using two-way ANOVA with the two factors being diet (STD and HFD) and treatment (vehicle and daidzein), followed by Bonferroni *post hoc* test for multiple comparisons. P<0.05 was considered to be significant.

## Results

### Effect of Diet and Subchronic Treatment with Daidzein on Cumulative Food/caloric Intake and Body Weight Gain

**Figure 1:**
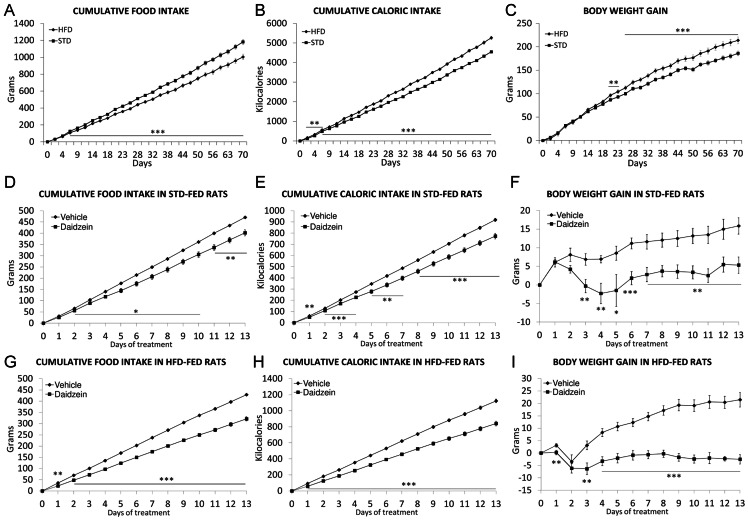
Effect of STD, HFD and daidzein (50 mg kg^−1^ day^−1^ body weight) on cumulative food intake (A, D, G), caloric intake (B, E, H) and body weight gain (C, F, I) after 70 days of feeding and 13 days of treatment. During the experiment, animals were maintained with their respective diets, and food intake and body weight was recorded daily. Histograms represent the mean ± s.e.m. (*n* = 8–16). Two-way ANOVA and Bonferroni *post hoc* test: *P<0.05, **P<0.01, ***P<0.001 *vs.* STD-fed group.

To establish the effect of both diets on energy balance, we first measured cumulative food/caloric intake and body weight gain on the rats fed STD and HFD over the first 10 weeks of the feeding period (Fig. 1). Rats fed STD significantly accumulated more food intake than rats fed HFD from the seventh day on (*P*<0.001) (Fig. 1A). The cumulative caloric intake was higher in HFD than STD group, and such differences were statistically significant from the second day on (between second and sixth days, *P*<0.01; seventh day on, *P*<0.001) (Fig. 1B). Differences in body weight gain were consistent with those of cumulative caloric intake between STD and HFD (Fig. 1C). Body weight gain showed a significant divergence between rats fed both diets from the third week on (day 21–23, *P*<0.01; day 25 on, *P*<0.001). Two-way ANOVA analysis showed a significant diet effect on cumulative food intake (Diet: *F*_1,900_ = 3092.56, *P*<0.0001), cumulative caloric intake (Diet: *F*_1,900_ = 447.53, *P*<0.0001) and body weight gain (Diet: *F*_1,900_ = 130.75, *P*<0.0001).

After 10 weeks of feeding, rats received a daily dose of either vehicle (10% Tocrisolve in saline) or daidzein (50 mg kg^−1^ body weight) during 13 days, while the diets remained unchanged (Fig. 1). We observed that treatment with daidzein reduced significantly cumulative food intake in rats fed STD and HFD from the day 1–2 of treatment on (Fig. 1D,G). In both groups, daidzein also reduced significantly cumulative caloric intake from the first day of treatment (Fig. 1E,H). These results also correlated with a significant decrease in body weight gain from the third day of treatment in STD-fed rats, and from the first day of treatment in HFD-fed rats (Fig. 1F,I). Two-way ANOVA analysis showed that treatment induced a significant effect on cumulative food intake (*F*_1,196_ = 48.27, *P*<0.0001 for STD; *F*_1,196_ = 76.69, *P*<0.0001 for HFD), cumulative caloric intake (*F*_1,196_ = 133.89, *P*<0.0001 for STD; *F*_1,196_ = 34.33, *P*<0.0001 for HFD) and body weight gain (*F*_1,196_ = 92.08, *P*<0.0001 for STD; *F*_1,196_ = 130.36, *P*<0.0001 for HFD).

### Effect of Diet and Treatment with Daidzein on Glucose Tolerance

**Figure 2:**
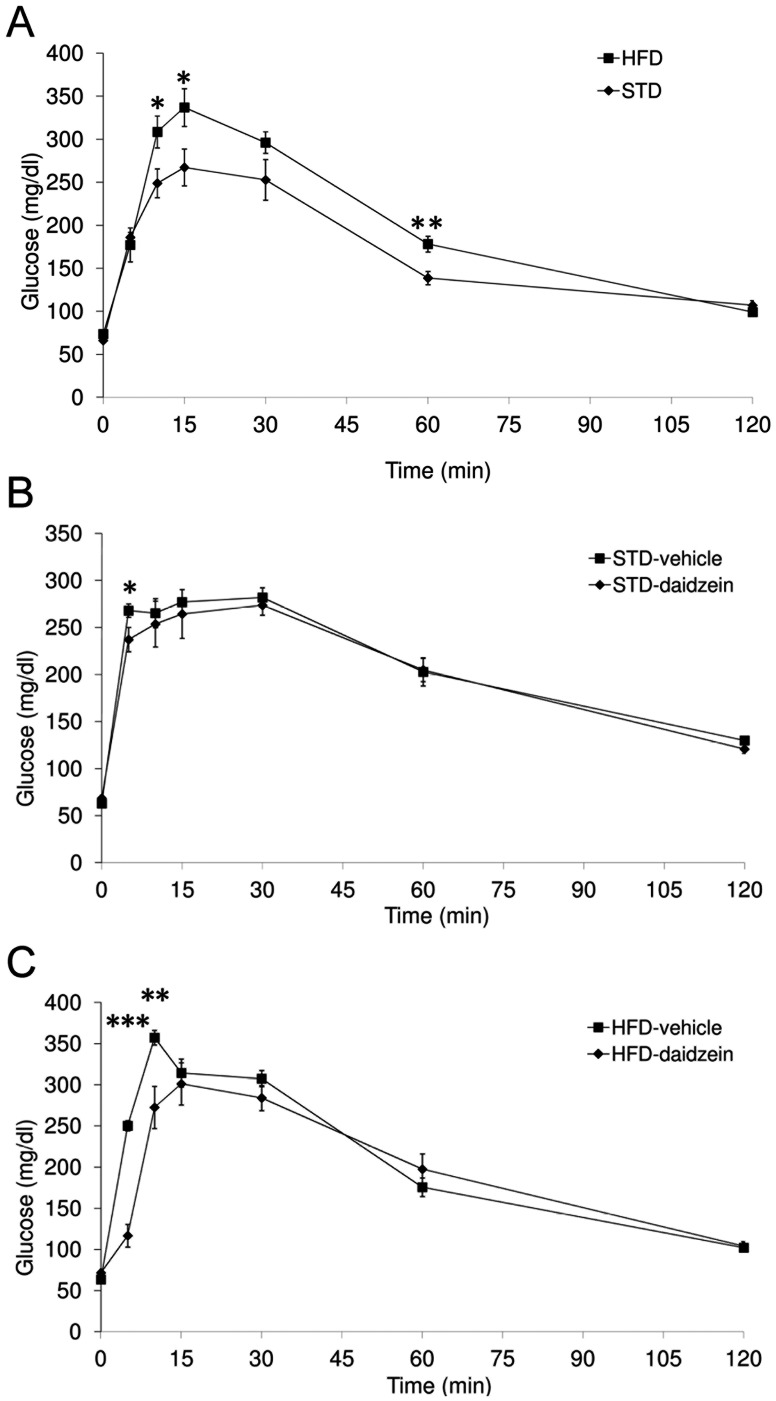
Effect of standard and high fat diets on glucose tolerance in fasted rats fed for 10 weeks or after repeated administration with daidzein (50 mg kg^−1^ day^−1^ body weight). Blood glucose levels were evaluated before (0 min) and after (5, 10, 15, 30, 45, 60 and 120 min) glucose overload (2 mg/kg body weight). Points indicate the mean ± s.e.m. (*n* = 8). Two-way ANOVA and Bonferroni *post-hoc* test: *P<0.05, **P<0.01, ***P<0.001 *vs.* STD-fed rats or vehicle-treated group.

We checked the glucose tolerance in rats exposed to a STD or HFD before (10^th^ week of feeding) and during daidzein treatment (5^th^ day of treatment). Glucose tolerance differed significantly between HFD and STD-fed rats (diet effect: *F*_1,98_ = 14.29, *P* = 0.0003), which was significant at 10, 15 and 60 minutes after glucose load (Fig. 2A).

Daidzein did not induce changes in glucose tolerance of STD-fed rats when compared with those treated with the vehicle (treatment effect: *F*_1,98_ = 1.67, *P* = 0.1999), only a lower level of glucose was detected at 5 minutes after glucose load (Fig. 2B). In contrast, daidzein produced a significant improvement of glucose tolerance in rats fed HFD (treatment effect: *F*_1,98_ = 17.92, *P*<0.0001), which was evident at 5 and 10 minutes after glucose load (Fig. 2C).

### Effect of Diet and Subchronic Treatment with Daidzein on Plasma Metabolites and Hormone Levels

To further evaluate the effects of the diets and daidzein treatment on energy balance, we analyzed several metabolites and metabolic hormones in plasma two hours after the last dose of daidzein or vehicle. After two-way ANOVA analysis, results showed that diet produced a significant effect in the circulating levels of triglycerides (*F*_1,28_ = 10.78, *P* = 0.0028), total cholesterol (*F*_1,28_ = 25.21, *P*<0.0001) and HDL-cholesterol (*F*_1,28_ = 16.3, *P* = 0.0004), that is, an increase of triglycerides, total and HDL-cholesterol in HFD-fed rats compared with the the STD group (*P*<0.05) (Table 1). No effect of treatment and interaction were detected in the circulating levels of triglycerides and total cholesterol. However, we observed a treatment effect on HDL-cholesterol (*F*_1,28_ = 4.6, *P* = 0.0416), which was related to a decrease in the plasma levels of daidzein-treated STD-fed rats (*P*<0.05). No interaction between factors was found. We did not detected changes in plasma levels of glucose (Table 1).

**Table 1:** Effect of daidzein on plasma metabolistes and hormones of STD and HFD-fed rats^1^.

	STD-vehicle	STD-daidzein	HFD-vehicle	HFD-daidzein
**Glucose (md/dL)**	154.13±11.6	148.6±10.6	184.5±17.5	148.6±5.8
**Triglycerides (mg/dL)**	109±12.4	105.1±11.02	174.5±16.8**	139.7±19.2
**Total cholesterol (mg/dL)**	67.4±6.2	59.6±2.6	86.2±3.4 *	83±3.7
**HDL-cholesterol (mg/dL)**	27.1±1.1	24.2±0.8 *	31.1±1.5 *	29.2±0.8
**Insulin (ng/mL)**	2.96±0.4	1.55±0.2*	5.02±0.5*	2.64±0.6**^#^**
**Leptin (pg/mL)**	1636±546	9217±1650**	1690±230	8329±2229**^#^**
**Adiponectin (ng/mL)**	4187±536	2602±217*	5603±308*	3457±289**^###^**
**Testosterone (ng/mL)**	1.93±0.2	1.49±0.1	2.41±0.2	1.78±0.1
**17β-estradiol (pg/mL)**	202.1±7.7	227.8±12.1	199.2±10.4	241.8±4.5**^##^**

1 Levels of plasma metabolites and hormones in rats fed STD or HFD and treated with vehicle or daidzein (50 mg/kg). Values represent the mean ± s.e.m. (*n* = 8). Two-way ANOVA and Bonferroni post test for multiple comparisons: *P<0.05, **P<0.01 *vs.* STD rats treated with vehicle; ^#^ *P*<0.05, ^##^ *P*<0.01, ^###^ *P*<0.001 *vs.* HFD rats treated with vehicle. Diet, treatment and interaction effects are described in the text.

Two-way ANOVA analysis showed that diet produced a significant effect on plasma levels of insulin (*F*_1,28_ = 9.19, *P* = 0.0052) and adiponectin (*F*_1,28_ = 10.06, *P* = 0.0037), but not in plasma levels of leptin, testosterone and 17β-estradiol (Table 1). HFD-fed rats showed increased levels of insulin and adiponectin compared with STD-fed rats (*P*<0.05). Treatment with daidzein induced significant effects on plasma levels of insulin (*F*_1,28_ = 13.23, *P* = 0.0011), leptin (*F*_1,22_ = 29.25, *P*<0.0001), adiponectin (*F*_1,28_ = 27.15, *P*<0.0001), testosterone (*F*_1,28_ = 5.43, *P* = 0.0272) and 17β-estradiol (*F*_1,26_ = 12.73, *P* = 0.0014). Bonferroni test indicated that insulin and adiponectin were decreased and leptin was increased in daidzein-treated rats fed STD and HFD. Finally, daidzein only produced a significant increase of 17β-estradiol in the plasma of HFD-fed rats (Table 1). No interaction between factors was found in the hormones analyzed.

### Newborn Cells in Subgranular Zone of Dentate Gyrus

**Figure 3:**
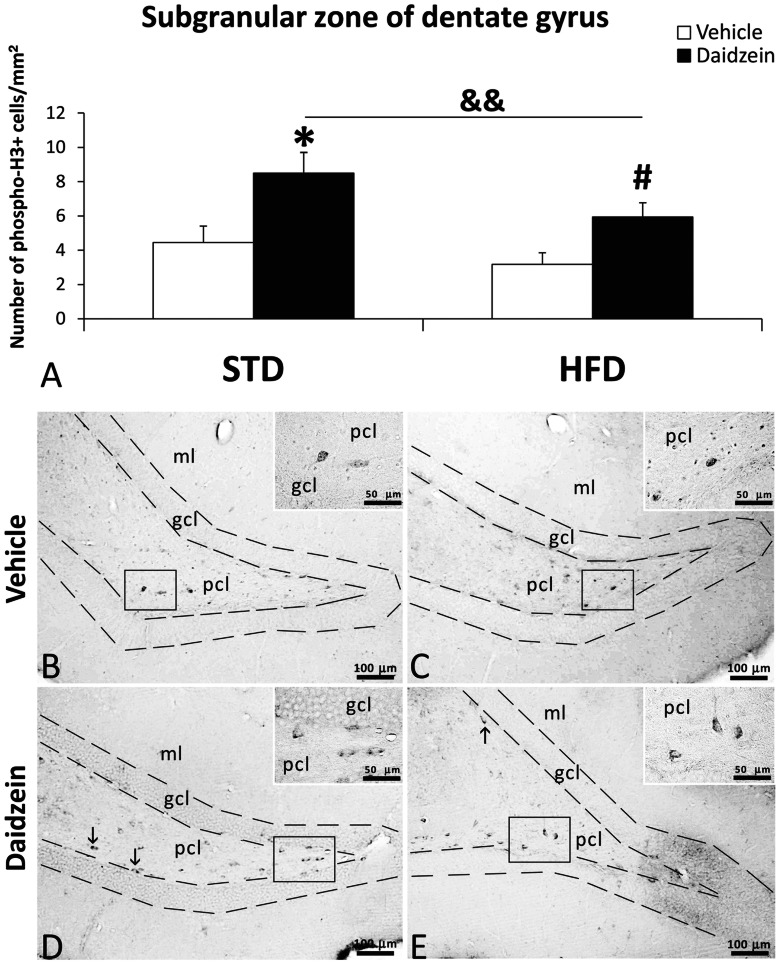
Effect of daidzein on mitosis in the SGZ of DG in rats fed STD and HFD by phospho-histone H3 immunohistochemistry. **A**) The number of positive cells is significantly greater in STD and HFD rats treated with daidzein. **B–E**) Representative microphotographs show low- and high- magnification (**insets**) views of the typical mitotic cells at the proximal border of the granular cell layer (**arrows**). The histogram represents the mean ± s.e.m. per area (mm^2^) (*n* = 6–8) of phospho-H3-ir cells per experimental group. Two-way ANOVA: ^&&^ *P*<0.01 for treatment effect (vehicle *vs.* daidzein). Bonferroni *post* test: *P<0.05 *vs.* STD-fed rats treated with vehicle, ^#^ *P*<0.05 *vs.* HFD-fed rats treated with vehicle. Scale bars are included in each image.

**Figure 4:**
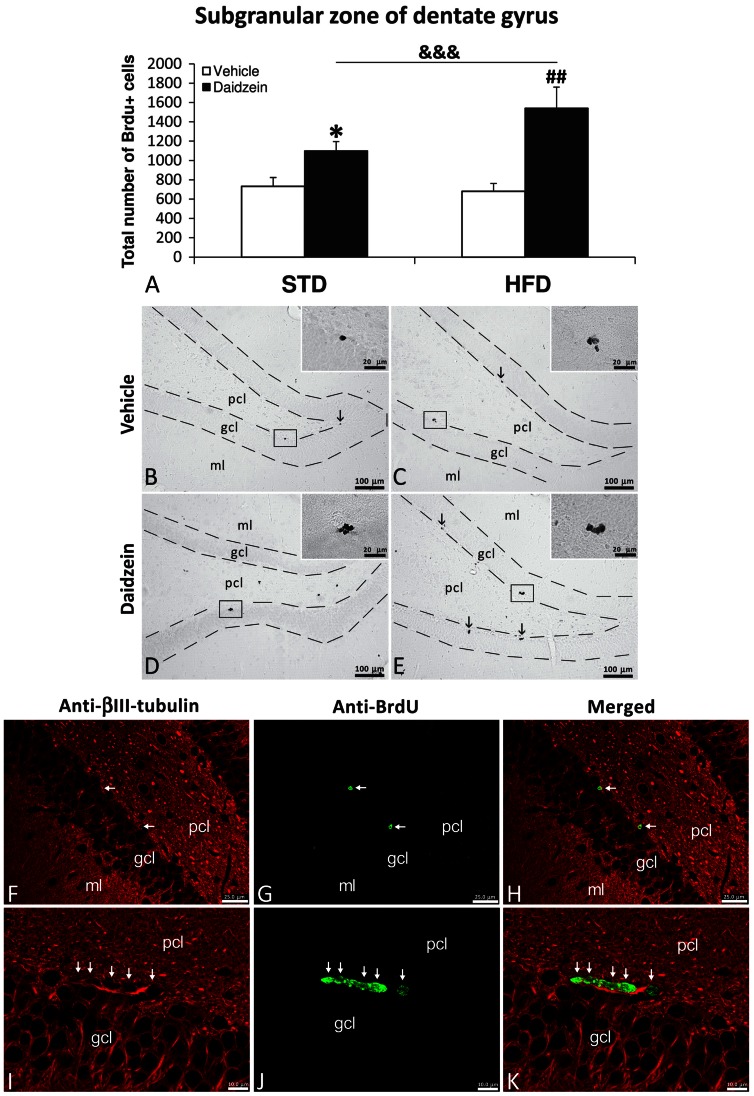
Effect of daidzein on cell proliferation in the SGZ of DG in rats fed STD and HFD by BrdU immunohistochemistry. **A**) The number of positive cells is significantly greater in STD and HFD rats treated with daidzein. **B–E**) Representative microphotographs show low- and high- magnification (**insets**) views of the typical clustering of newborn cells at the inner border of the granular cell layer (**arrows**). **F-K**) Newborn cells in the SGZ showing the co-localization of the neuron-specific β3-tubulin (red) and BrdU (green) by immunofluorescence. The histogram represents the mean ± s.e.m. (*n* = 6–8) of the total number of BrdU-ir nuclei per experimental group. Two-way ANOVA: ^&&&^ *P*<0.001 for treatment effect (vehicle *vs.* daidzein). Bonferroni *post* test: *P<0.05 *vs.* STD-fed rats treated with vehicle, ^##^ *P*<0.01 *vs.* HFD-fed rats treated with vehicle. Scale bars are included in each image.

To investigate the impact of both diets and daidzein treatment on hippocampal cell proliferation, we evaluated newborn cells in the SGZ of the DG by the analysis of the mitosis-related protein phospho-histone H3 and after 3 days of BrdU administration (50 mg kg^−1^). No differences in the number of phosphor-H3-ir cells were detected between STD and HFD diets (Fig. 3A), but daidzein-treated rats displayed a higher number of phosphor-H3-ir cells in SGZ compared with those detected in vehicle-treated rats in both STD and HFD diets (STD-vehicle: 4.45±0.96 *vs.* STD-daidzein: 8.49±1.21, *P*<0.05; HFD-vehicle: 3.38±0.91 *vs.* HFD-daidzein: 5.73±1.24, *P*<0.05) (Fig. 3). No differences in the number of BrdU-ir cells were detected between STD and HFD diets (Fig. 4A). Also, daidzein-treated rats displayed a higher number of BrdU-ir cells in SGZ compared with those detected in vehicle-treated rats in both STD and HFD diets (STD-vehicle: 16.29±3.10 *vs.* STD-daidzein: 24.44±2.14, *P*<0.05; HFD-vehicle: 15.13±2 *vs.* STD-daidzein: 34.25±5.23, *P*<0.01), showing an increase of approximately 50% and 126% respectively (Fig. 4A). Two-way ANOVA showed a significant treatment effect, indicating that the number of phosphor-H3 and BrdU-ir cells was affected by daidzein in STD and HFD-fed rats (phosphor-H3: *F*_1,28_ = 8.52, *P*<0.01; BrdU: *F*_1,22_ = 14.66, *P*<0.001). No effects of diet were seen in the number of phosphor-H3 and BrdU-ir cells and no interaction between diet and daidzein was detected.

To address whether newborn cells were programmed to become to putative neurons after 3 days of BrdU administration, we used a specific cell marker such as β3-tubulin (found almost exclusively in neurons). We found newborn BrdU-ir cells that expressed the neuron-specific β3-tubulin in the SGZ of the four experimental groups (Fig. 4E–K).

### Effect of Diet and Subchronic Treatment with Daidzein on Apoptotic Cells in Hippocampus

**Figure 5:**
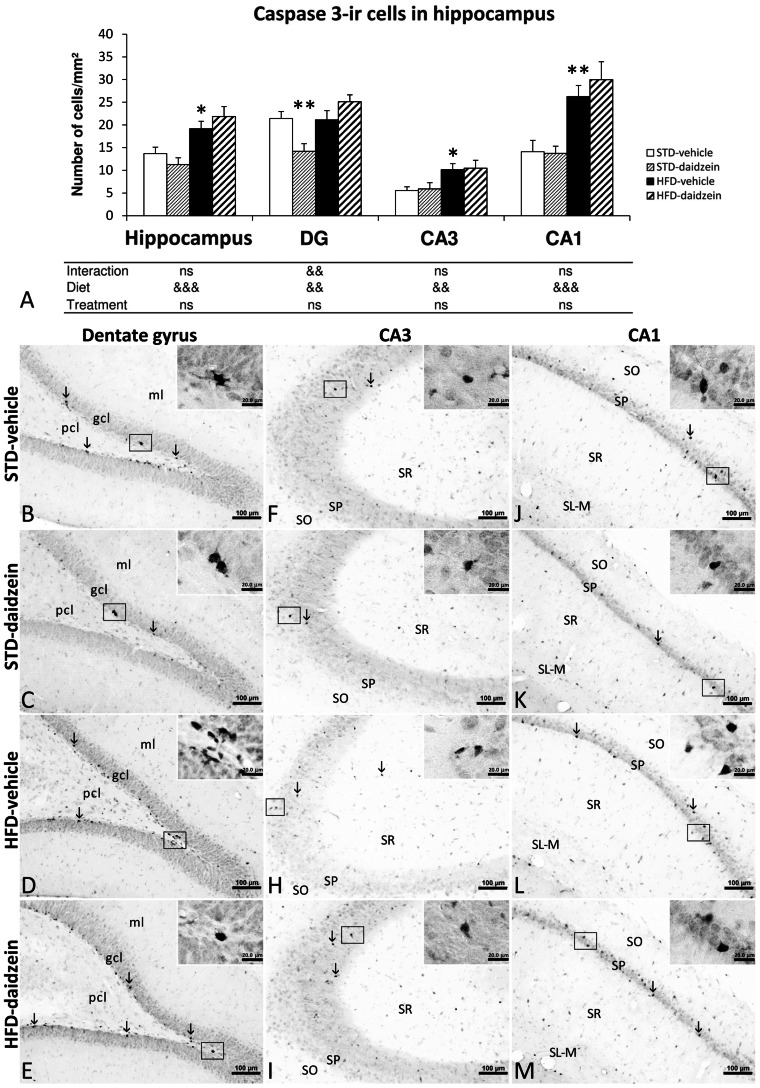
Effect of daidzein on proapoptosis in the hippocampus of rats fed STD and HFD by caspase-3 immunohistochemistry. **A**) The histogram represents the mean ± s.e.m. per area (mm^2^) of caspase 3-ir cells (*n* = 8). The number of positive cells is significantly greater in the hippocampi of rats fed HFD, but it is especially lower in the dentate gyrus of STD-fed rats treated with daidzein. **B–M**) Representative microphotographs show low- and high- (**insets**) magnification view of caspase 3-ir cells in the dentade gyrus, CA3 and CA1 of the hippocampus (**arrows**). Two-way ANOVA: ^&&^ *P*<0.01, ^&&&^ *P*<0.001 for diet effect (STD *vs.* HFD) or interaction (treatment *vs.* diet). Bonferroni *post* test: *P<0.05, **P<0.01 *vs.* STD-fed rats treated with vehicle. Scale bars are included in each image.

**Figure 6:**
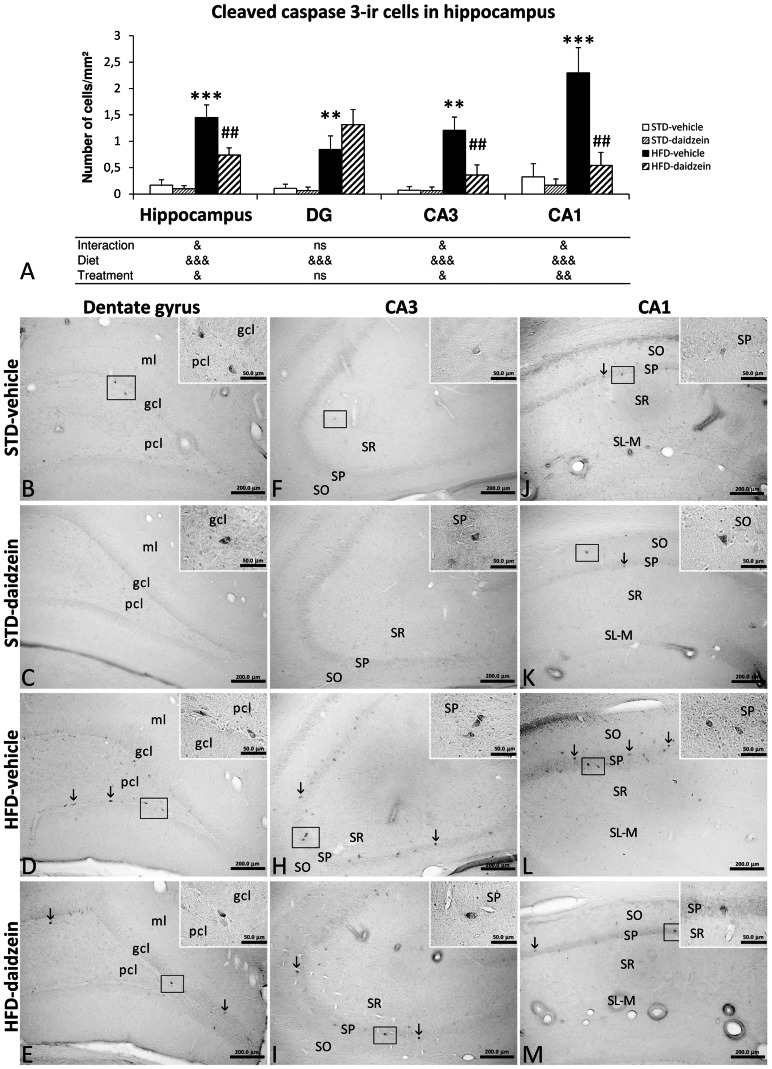
Effect of daidzein on apoptosis in the hippocampus of rats fed STD and HFD by cleaved caspase-3 immunohistochemistry. **A**) The histogram represents the mean ± s.e.m. per area (mm^2^) of cleaved caspase 3-ir cells (*n* = 8). The number of positive cells is significantly greater in the whole hippocampi of rats fed HFD, but it is especially lower in the DG of STD-fed rats treated with daidzein. **B–M**) Representative microphotographs show low- and high- (**insets**) magnification view of cleaved caspase 3-ir cells in the dentade gyrus, CA3 and CA1 of the hippocampus (**arrows**). Two-way ANOVA: ^&^ *P*<0.05, ^&&^ *P*<0.01, ^&&&^ *P*<0.001 for diet effect (STD *vs.* HFD), treatment effect (vehicle *vs*. daidzein) or interaction (treatment *vs.* diet). Bonferroni *post* test: **P<0.01, ***P<0.001 *vs.* STD-fed rats treated with vehicle, ^##^ *P*<0.01 *vs.* HFD-fed rats treated with vehicle. Scale bars are included in each image.

Proapoptotic and apoptotic cells, as determined by caspase-3 and cleaved caspase-3 immunostaining respectively, were assessed in DG, CA3 and CA1 areas of hippocampus (Figs. 5 and 6). Two-way ANOVA showed that diet induced a significant effect in the number of proapoptotic caspase 3-ir cells in all hippocampal areas analyzed (Total hippocampus: *F*_1,27_ = 22.83, *P*<0.001; DG: *F*_1,27_ = 9.43, *P*<0.01; CA3: *F*_1,27_ = 11.94, *P*<0.01; CA1: *F*_1,27_ = 28.21, *P*<0.01). Bonferroni *post* test indicated that HFD induced an increase in the number of caspase 3-ir cells in the CA3 and CA1 areas (*P*<0.05; *P*<0.01, respectively), and consequently in total hippocampus (*P*<0.05). Bonferroni *post* test showed that daidzein treatment induced a specific decrease of caspase 3-ir cells in the DG of rats fed STD (*P*<0.01). No significant differences in proapoptotic cells were found between HFD-vehicle and HFD-daidzein groups in the hippocampal regions analyzed (Fig. 5). We only observed interaction between diet and treatment in the DG (*F*_1,27_ = 9.43, *P*<0.01), i.e., daidzein produced opposite effects in the number of proapoptotic caspase 3-ir cells in a diet-dependent manner.

Regarding cleaved caspase-3 immunostaining (Fig. 6), two-way ANOVA showed that diet also induced a significant effect in the number of apoptotic cleaved caspase 3-ir cells in all hippocampal areas analyzed (Total hippocampus, DG, CA3 and CA1: *F*_1,28_>15.04, *P*<0.001). Bonferroni *post* test indicated that HFD also induced an increase in the number of cleaved caspase 3-ir cells in the DG, CA3 and CA1 areas (*P*<0.01; *P*<0.01; *P*<0.001 respectively), and consequently in total hippocampus (*P*<0.001). Interestingly, we observed a daidzein effect in CA3 and CA1, but not in DG (Total hippocampus: *F*_1,28_ = 6.74, *P*<0.05; DG: *F*_1,28_ = 1.15, *P* = 0.29; CA3: *F*_1,27_ = 6.76, *P*<0.05; CA1: *F*_1,28_ = 9.96, *P*<0.01). Thus, Bonferroni *post* test showed that daidzein treatment induced a decrease of cleaved caspase 3-ir cells in the CA3 and CA1 of rats fed HFD (*P*<0.01). No significant differences in apoptotic cells were found between STD-vehicle and STD-daidzein groups in the hippocampal regions analyzed (Fig. 6). We observed interaction between diet and treatment in the CA3 and CA1 (CA3: *F*_1,28_ = 9.60, *P*<0.05; CA1: *F*_1,28_ = 6.97, *P*<0.05), i.e., daidzein produced opposite effects in the number of apoptotic cleaved caspase 3-ir cells in a diet-dependent manner.

### Effect of Diet and Subchronic Treatment with Daidzein on FosB Cells in Hippocampus

**Figure 7:**
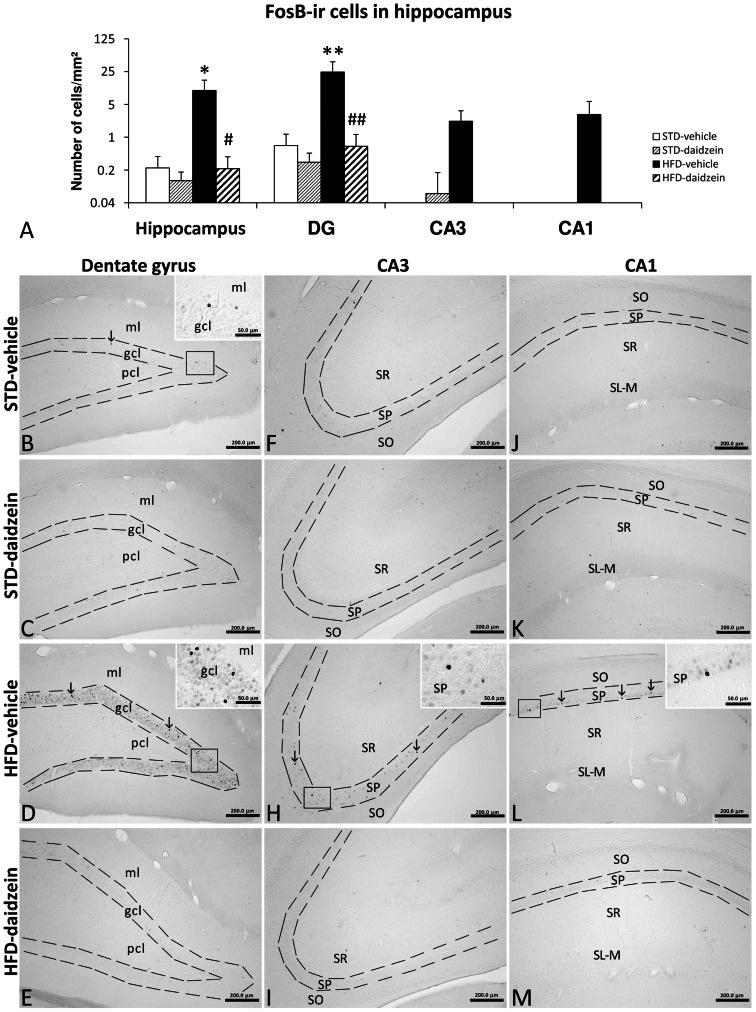
Effect of daidzein on the food reward factor FosB in the hippocampus of rats fed STD and HFD. **A**) The histogram represents the mean ± s.e.m. per area (mm^2^) of FosB-ir nuclei (*n* = 8). **B–M**) Representative microphotographs show low- and high- (**insets**) magnification view of FosB-ir cells in the dentade gyrus, CA3 and CA1 of the hippocampus (**arrows**). Bonferroni *post* test: *P<0.05, **P<0.01 *vs.* STD-fed rats treated with vehicle, ^#^ *P*<0.05, ^##^ *P*<0.01 *vs.* HFD-fed rats treated with vehicle. Scale bars are included in each image.

We also investigated the induction of FosB as a brain reward factor that is influenced by susceptibility to overeating and weight gain (Fig. 7). After two-way ANOVA, no significant diet and treatment effects or interaction were found, but Bonferroni *post* test showed that HFD induced a prominent increase of FosB-ir cells in the DG (*P*<0.01) and the presence of labeled cells in the CA3 and CA1. Interestingly, daidzein treatment in the HFD-fed rats produced a decrease of the number of FosB-ir cells in the DG (*P*<0.01) and the non-presence of labeled cells in CA3 and CA1. We hardly detected any FosB-ir cells in the hippocampus of STD-fed rats (Fig. 7).

### Effect of Diet and Subchronic Treatment with Daidzein on Gliosis in Hippocampus

**Figure 8:**
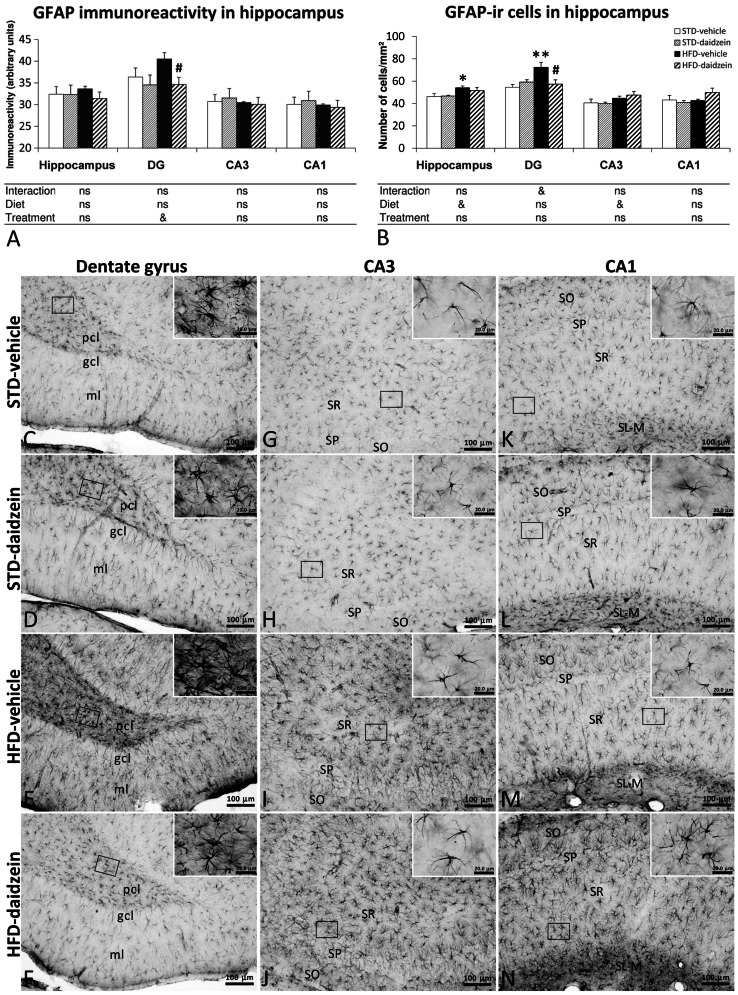
Effect of daidzein on astrogliosis in the hippocampus of rats fed with STD and HFD by GFAP immunohistochemistry. **A, B**) The histograms represent the mean ± s.e.m. of GFAP immunoreactivity and the mean ± s.e.m. per area (mm^2^) of GFAP-ir cells (*n* = 8). The number of positive cells is significantly higher in the hippocampi of HFD-fed rats, and especially lower in the dentate gyrus of daidzein-treated, HFD-fed rats. **C-M**) Representative microphotographs show low- and high- (**insets**) magnification view of GFAP-ir cells in the dentade gyrus, CA3 and CA1 of the hippocampus. Two-way ANOVA: ^&^ *P*<0.05 for diet effect (STD *vs.* HFD) or interaction (treatment *vs.* diet). Bonferroni *post* test: *P<0.05, **P<0.01 *vs.* STD-fed rats treated with vehicle; ^#^ *P*<0.05 *vs.* HFD-fed rats treated with vehicle. Scale bars are included in each image.

**Figure 9:**
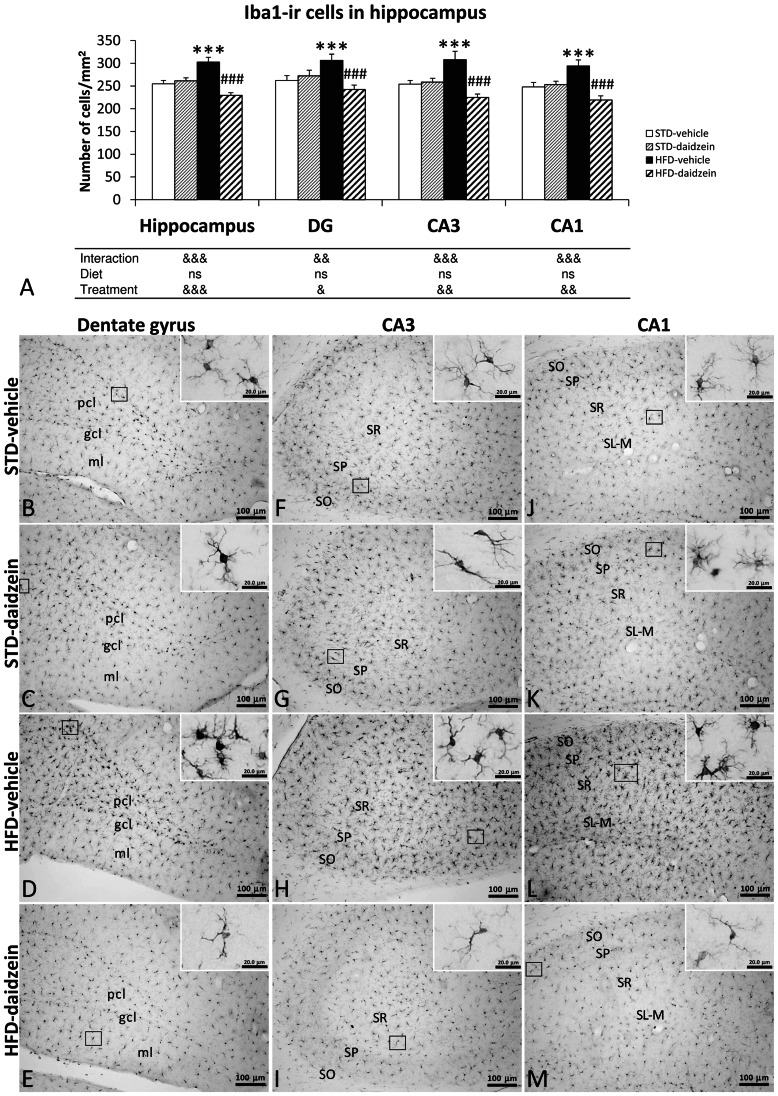
Effect of daidzein on microgliosis in the hippocampus of rats fed with STD and HFD by Iba1 immunohistochemistry. **A)** The histogram represents the mean ± s.e.m. per area (mm^2^) of Iba1-ir cells (*n* = 8). The number of positive cells is significantly higher in the hippocampi of HFD-fed rats, but especially lower in the hippocampi of HFD-fed rats after daidzein teratment. **B–M**) Representative microphotographs show low- and high- (**insets**) magnification view of Iba1-ir cells in the dentade gyrus, CA3 and CA1 of the hippocampus. Two-way ANOVA: ^&^ *P*<0.05, ^&&^ *P*<0.01 for diet effect (STD *vs.* HFD) or interaction (treatment *vs.* diet). Bonferroni *post* test: *P<0.05, **P<0.01 *vs.* STD-fed rats treated with vehicle; ^#^ *P*<0.05 *vs.* HFD-fed rats treated with vehicle. Scale bars are included in each image.

To investigate the impact of the diets and daidzein treatment on hippocampal gliosis, we evaluated the GFAP immunoreactivity, the number of astrocytes that express GFAP and the number of microglia that express Iba1 (Figs. 8 and 9). Two-way ANOVA showed that treatment induced an effect on GFAP expression in the DG (*F*_1,23_ = 4.19, *P*<0.05), showing, by Bonferroni *post* test, a specific decrease of GFAP immunoreactivity in DG of HFD-fed rats treated with daidzein (*P*<0.05) (Fig. 8A). Diet effect and interaction were not detected when GFAP immunoreactivity was analyzed. In contrast, two-way ANOVA showed that diet induced an effect on astrogliosis in the whole hippocampus (*F*_1,23_ = 4.97, *P*<0.05), but specifically in CA3 (*F*_1,23_ = 4.26, *P*<0.05) (Fig. 8B). Bonferroni *post* test indicated a specific increase in the number of GFAP-ir cells only in DG of HFD-fed rats (*P*<0.01) and a specific reduction in the number of GFAP-ir cells only in DG of HFD-fed rats treated with daidzein (*P*<0.05). No significant changes were detected in the hippocampus of STD-fed rats treated with daidzein. As a consequence, two-way ANOVA showed an interaction between diet and treatment factors in DG (*F*_1,23_ = 4.8, *P*<0.05), i.e., daidzein produced different effects in astrogliosis in a diet-dependent manner (Fig. 8B).

Regarding Iba1-ir cells (Fig. 9), two-way ANOVA showed that diet did not induce any effect, but Bonferroni *post* test indicated a significant increase in the number of Iba1-ir cells in the hippocampus of HFD-fed rats (*P*<0.001). We detected an important treatment effect in all hippocampal regions analyzed (Total hippocampus: *F*_1,28_ = 17.74, *P*<0.001; DG: *F*_1,28_ = 5.10, *P*<0.05; CA3: *F*_1,28_ = 11.16, *P*<0.01; CA1: *F*_1,28_ = 11.56, *P*<0.01). Thus, the hippocampi of daidzein-treated HFD-fed rats showed a prominent reduction of the number of Iba1-ir cells (*P*<0.001). As a consequence, we observed interaction between diet and treatment in all hippocampal areas (Total hippocampus: *F*_1,28_ = 25.45, *P*<0.0001; DG: *F*_1,28_ = 9.79, *P*<0.01; CA3: *F*_1,28_ = 13.78, *P*<0.001; CA1: *F*_1,28_ = 15.22, *P*<0.001)., i.e., daidzein produced different effects in the number of Iba1-ir cells in a diet-dependent manner (Fig. 9).

### Effect of Diet and Subchronic Treatment with Daidzein on Estrogen Receptor Alpha Immunoreactivity in Hippocampus

**Figure 10:**
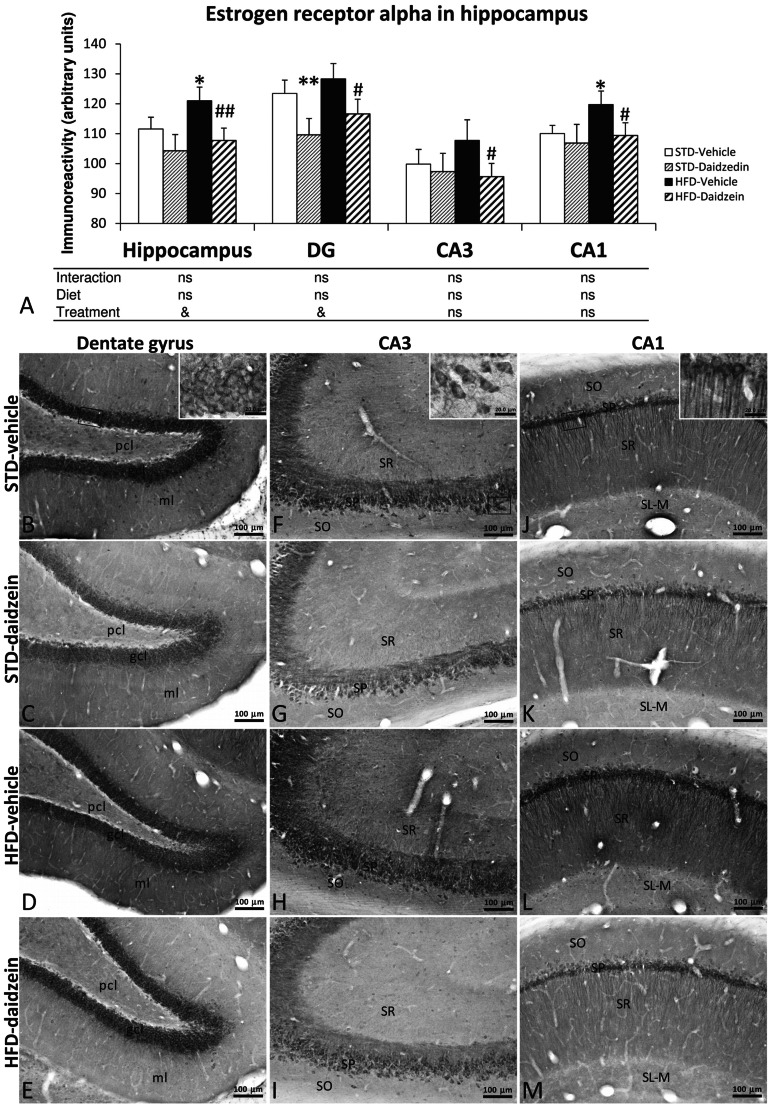
Effect of daidzein on the immunohistochemical expression of estrogen receptor alpha (ERα) in the hippocampus of rats fed with STD and HFD. **A**) The histogram represents the mean ± s.e.m. (*n* = 8). ERα immunoreactivity is higher in the hippocampi of rats fed HFD, but it is significantly reduced after daidzein treatment. **B–M**) Representative microphotographs show low- and high- (**insets**) magnification view of ERα immunoreactivity in the dentade gyrus, CA3 and CA1 of the hippocampus. Two-way ANOVA: ^&^ *P*<0.05 for treatment effect (vehicle *vs.* daidzein). Bonferroni *post* test: *P<0.05, **P<0.01 *vs.* STD-fed, vehicle-treated rats; ^#^ *P*<0.05, ^##^ *P*<0.01 *vs.* HFD-fed, vehicle-treated rats. Scale bars are included in each image.

Representative microphotographs of estrogen receptor alpha (ERα) immunoreactivity in DG, CA3 and CA1 corresponding to the four experimental groups are shown in Figure 10. An intense ERα immunoreactivity was detected in the somata of the granular cells of DG, and in the somata and proximal dendrites of the pyramidal neurons from CA3 to CA1 (Fig. 10B–E). Some ERα-ir neurons were also observed in the pcl and ml of the DG, and in the SO and SR of CA areas (Fig. 10F-M).

Results corresponding to the quantification of ERα immunoreactivity are shown in Figure 10A. Two-way ANOVA showed no significant effect of diet when analyzing ERα immunoreactivity in the total hippocampus or in its different areas. However, Bonferroni *post* test indicated that HFD induced an increase of ERα expression in total hippocampus (*P*<0.05), and this increase was specific in CA1 area only (*P*<0.05). Two-way ANOVA analysis showed that daidzein treatment induced a significant effect on ERα expression in the total hippocampus (*F*_1,28_ = 5.05, *P*<0.05), being more evident in DG (*F*_1,28_ = 6.49, *P*<0.05). Bonferroni *post* test indicated that daidzein effect is mainly due to a significant decrease in all hippocampal regions analyzed of HFD-fed rats (all at *P*<0.05). In addition, daidzein only induced a decrease of ERα immunoreactivity in DG of STD-fed rats (*P*<0.01). No significant interaction between diet and treatment was detected, i.e., daidzein produced the same effect in ERα expression throughout the hippocampus, regardless of the diet.

## Discussion

In the present study we analyzed the neuroprotective role of daidzein (50 mg kg^−1^ body weight) in rats fed a very high fat diet (60% fat) as a brain insult model. The dose was selected on the basis of the acute effects of this isoflavone on a feeding test [4]. The main effects observed are summarized in Table 2. A high fat feeding was performed for 12 weeks in order to induce metabolic impairments in comparison with standard feeding. So, after 12 weeks, HFD-fed rats displayed overweight, glucose intolerance and high plasma levels of triglycerides and cholesterol when were compared with STD-fed rats. Additionally, the highest plasma levels of insulin and adiponectin were found in HFD-fed rats. Interestingly, STD and HFD-fed rats did not display differences in the number of newborn cells in the SGZ of dentade gyrus. However, HFD-fed rat hippocampi showed an increased number of caspase-3, FosB, GFAP and Iba1-ir cells, and higher ERα immunoreactivity. After producing important metabolic and hippocampal alterations in this experimental model of high-fat feeding, we demonstrated that pharmacological doses (50 mg kg^−1^ body weight) of the isoflavone daidzein induced increased newborn cells, showing a neuronal phenotype (β3-tubulin+), and reduced apoptosis and gliosis in the hippocampus of HFD-fed rats. These effects were associated to a reduction of food/caloric intake and body weight gain, an improvement of glucose tolerance, a decrease of plasma levels of cholesterol, insulin, adiponectin and testosterone and an increase of plasma levels of leptin and 17β-estradiol. In a previous study of our group we showed that the reduction of body weight after a subchronic treatment of daidzein was accompanied with a decrease of fat content in the liver [4]. These results were associated with high leptin levels and low adiponectin content in plasma. Additionally, adipose tissue and liver displayed marked changes induced by daidzein that affect transcription factors and lipogenic enzymes, especially SCD1, a pivotal enzyme in obesity. UCP1, an important enzyme involved in thermogenesis, was increased in brown adipose tissue after daidzein treatment [4].

**Table 2:** Summary of the effects produced by diet, treatment and interaction on each parameter analyzed^1^.

	Diet effect	Treatment effect	Interaction
	STD *vs*. HFD	Vehicle *vs*. daidzein	Diet *vs*. Treatment
**Food intake**	*** ↓	*** ↓	ns
**Caloric intake**	*** ↑	*** ↓	ns
**Body weight**	*** ↑	*** ↓	ns
**Glucose**	Ns	Ns	ns
**Triglycerides**	** ↑	Ns	ns
**Total cholesterol**	*** ↑	Ns	ns
**HDL-cholesterol**	*** ↑	* ↓	ns
**Insulin**	** ↑	** ↓	ns
**Leptin**	Ns	*** ↑	ns
**Adiponectin**	** ↑	*** ↓	ns
**Testosterone**	Ns	* ↓	ns
**17β-estradiol**	Ns	** ↑	ns
**Phospho-H3**	Ns	** ↑	ns
**BrdU**	Ns	*** ↑	ns
**Total caspase-3**	*** ↑	Ns	ns
**Cleaved caspase-3**	*** ↑	* ↓	*
**GFAP**	* ↑	Ns	ns
**Iba1**	ns	*** ↓	***
**Estrogen receptor α**	ns	* ↑	ns

1 Diet effect (STD vs HFD), treatment effect (vehicle vs daidzein) and the interactions between both factors (diet vs treatment), analyzed by two-way ANOVA and Bonferroni post-test: ns means no statistical significance, *P<0.05; **P<0.01 and ***P<0.001. Arrows indicate the direction of the effect. For abbreviation see list.

It has been well documented that HFD impairs hippocampal neurogenesis [22]–[24]. For instance, Lindqvist et al. [23] demonstrated that just 4 weeks of feeding a diet rich in fat *ad libitum* (42% fat) decreased hippocampal neurogenesis. In contrast, Strandberg et al. [25] pointed out that ketogenic diets (80% fat) do not alter the neurogenesis in the rat dentate gyrus, suggesting that not only caloric intake but also other factors such as meal frequency, texture and content can make an impact on adult neurogenesis [13]. Despite of the glucose intolerance and the high levels of plasma triglycerides, total cholesterol and insulin in HFD-fed rats, no changes on SGZ cell proliferation were observed between STD and HFD rats. We suggest that this lack of difference in cell proliferation between STD and HFD rats could be related to the lack of significant differences in the plasma levels of basal glucose and leptin, indicating similar leptin resistance. Several studies suggest that high caloric diets rich in fat or cholesterol are environmental factors that can impede energy balance regulation and therefore influence the normal function of the brain [26]. Diet-induced obesity resulted in cognitive impairment whose mechanisms are associated with vascular damage to the central nervous system and numerous metabolic abnormalities including dyslipidaemia and glucose intolerance, producing functional resistance to insulin and leptin (diabetes) [27].

Data obtained from clinical, epidemiological and nutritional studies showed the beneficial effects of dietary phytoestrogens, including the estradiol (E2)-like soy isoflavone daidzein, on obesity and diabetes [28]. These improved metabolic consequences include glucose control, insulin resistance, and plasma lipid profile [29]–[31], as well as producing weight gain reduction in rats [32], [33]. Besides the modulatory role of the E2-like compounds on energy balance, gonadal hormones also play a significant role in the regulation of adult neurogenesis in the hippocampus [10] and neuroprotection after injury [34]. Pérez-Martín et al. [35] reported that ovariectomized animals treated with soy extract (60 mg kg^−1^ in drinking water) and estradiol (150 µg) for 10 weeks showed a significantly higher number of BrdU-immunoreactive cells in the SGZ. The present study demonstrates that a subchronic treatment of daidzein (50 mg kg^−1^) over 13 days had a similar effect on hippocampal cell proliferation, i.e., a significant increase in the number of BrdU-labeled cells in the SGZ of DG, being more prominent in rats fed HFD (approximately 50% *versus* 126% of increase in animals fed either STD or HFD respectively). Regarding the methodological disadvantage of BrdU labeling [36], this effect on cell proliferation was confirmed by the increase of phospho-H3-ir cells, a mitosis-related factor. The observed effect of daidzein, linked to both caloric intake reduction and the increase in SGZ cell proliferation, agrees with previous studies suggesting that a dietary restriction significantly enhances neuronal proliferation in the dentate gyrus of rats [37]. Considering this issue, we found that treatment of daidzein produced an increase of leptin levels in plasma in a diet-independent manner suggesting a link between the role of leptin in controlling appetite, intake reduction and cell proliferation. An interesting study of neonates treated with leptin showed increase in neurogenesis and survival of newborn cells in the hippocampus [38]. Furthermore, we showed that treatment with daidzein also induced an increase of E2 levels in plasma, being more prominent in HFD-fed rats. According to the literature [35], it is perfectly conceivable that the increase of plasma E2 induced by daidzein may be partly responsible for the enhancement of cell proliferation observed in the SGZ. An interesting hypothesis is that daidzein treatment increases estradiol secretion. In fact, daidzein is capable of promoting the production of estradiol in human trophoblast cells [39] and our data suggest that this effect is also present in our experimental animal model.

Further studies are needed to elucidate whether the effect of daidzein on hippocampal cell proliferation is produced via the participation of estrogen receptors (ERs). To give some light to this hypothesis we analyzed the immunohistochemical levels of the estrogen receptor alpha in the hippocampus of both STD and HFD-fed rats. ERα is involved in estradiol-induced enhancement of hippocampal neurogenesis [40]. Moreover, ERα has greater implication in energy metabolism [41]. Despite being no alteration of diet-dependent plasma E2 or testosterone levels, we found increased ERα immunoreactivity in the hippocampus of HFD-fed animals. Originally, the effects produced by E2 in the hippocampus have been attributed to gonadal estradiol that may reach the hippocampus via the blood-brain barrier [42]. However, hippocampal neurons express aromatase, the enzyme that converts testosterone to 17β-estradiol [43] and are capable of producing E2 *de novo* [44], [45] suggesting that part of the effects previously attributed to gonadal E2 may actually be caused by E2 which is cyclically produced and released within the hippocampus via regulation of E2 synthesis by gonadotropin-releasing hormones [46].

Daidzein treatment induced a decrease of the immunohistochemical expression of ERα in all hippocampal areas analyzed of HFD-fed animals, but only in the DG of STD-fed animal. This effect can be linked to the increased levels of plasma E2, being more prominent in HFD-fed rats. Similar effects of daidzein have been seen in other brain regions like bed nucleus of stria terminalis and medial amygdaloid nucleus [47]. Taking together these data, we can hypothesize that both daidzein treatment (E2-like compound) and the consequent increase of plasma E2 can cause receptor desensitization and a concomitant down-regulation of ERα expression in the hippocampus of HFD-fed rats. The effect of daidzein on cell proliferation linked to a reduction of ERα expression can be postulated by the fact that soy phytoestrogens are able to regulate estrogen receptor transcription [48] and estrogen receptor antagonism can block IGF1-induced hippocampal neurogenesis [49]. However, estrogen receptor-independent mechanisms cannot be excluded. Since we can’t exclude a possible direct hormonal effect of daidzein on hippocampal neurogenesis, further studies are needed to elucidate whether 1) the fat content of the diets affects the synthesis of 17β-estradiol in the hippocampus, 2) the rate of daidzein that crosses the blood brain barrier and 3) the role of estrogen receptors in proliferating cells.

Our data show that HFD increases the number of cells that express the apoptotic factor caspase-3, the food reward factor FosB and the glial-activation factors GFAP (astroglia) and Iba1 (microglia) in the hippocampus. These data suggest that a prolonged consumption of a diet with high content in fat may induce a reward response related to an overexpression of the transcription factor FosB that led to putative cell death and gliosis in the hippocampus, confirming the implication of this brain area in energy homeostasis [50]–[52]. It should be clarified that FosB is typically implicated in control of the reward system and development of drug addiction in the brain [53]. Estradiol and estrogen-like compounds, as daidzein, are powerful neuroprotective agents against numerous *in vivo* and *in vitro* apoptotic stimuli [54], [55]. Furthermore, these neuroprotective effects appear to utilize multiple mechanisms depending on the injury, including classical transcriptional signaling through estrogen receptors [5], [15], [56]. An interesting result from the present study was that daidzein administration, as well as the consequent increase of E2 level in plasma, affect the amount of caspase 3, FosB, GFAP and Iba1-ir cells in a diet-dependent manner. Thus, we observed a reduction of the number of caspase 3-ir cells in the DG of STD-fed rats, a reduction of GFAP-ir cells in the DG of HFD-fed rats and a decrease of FosB and Iba1-ir cells in all hippocampal areas analyzed of HFD-fed rats. Therefore, we can hypothesize a relationship between the increase of cell proliferation and the decrease of apoptosis and gliosis in the hippocampus of rats fed HFD and treated with daidzein.

In conclusion, the present study demonstrates that pharmacological administration of isoflavones can increase SGZ cell proliferation and reduce hippocampal apoptosis and gliosis in a metabolic context of HFD-induced obesity. The potential benefits derived from these actions further support for the incorporation of soy products to a healthy diet.

## Supporting Information

Figure S1 **Experimental design used for feeding, daidzein administration and 5-bromo-2′-deoxyuridine (BrdU) injections.** Animals were fed *ad libitum* for 84 days with two diets: a very high fat diet and a standard diet. After 70 days, when the weight curves achieved divergence and stabilization between both diets, rats received a daily intraperitoneal injection of daidzein (50 mg kg^−1^) or vehicle (Tocrisolve) for 13 days. Four days before sacrifice, animals received two daily intraperitoneal injection of BrdU (50 mg kg^−1^) at 10 hours intervals (8 a.m., 6 p.m.) for three consecutive days. Two hours after the last dose of daidzein, rats were sacrificed to collect blood samples and the brains.
(TIF)

Methodology S1 **Extensively description of methodology and antibodies used in the present study.**
(DOCX)
