# Supplementary figures and images for: Pharmacological Administration of the Isoflavone Daidzein Enhances Cell Proliferation and Reduces High Fat Diet-Induced Apoptosis and Gliosis in the Rat Hippocampus
Source: PLoS One. 2013 May 31;8(5):e64750. doi: 10.1371/journal.pone.0064750 (PMC3669353; doi:10.1371/journal.pone.0064750)

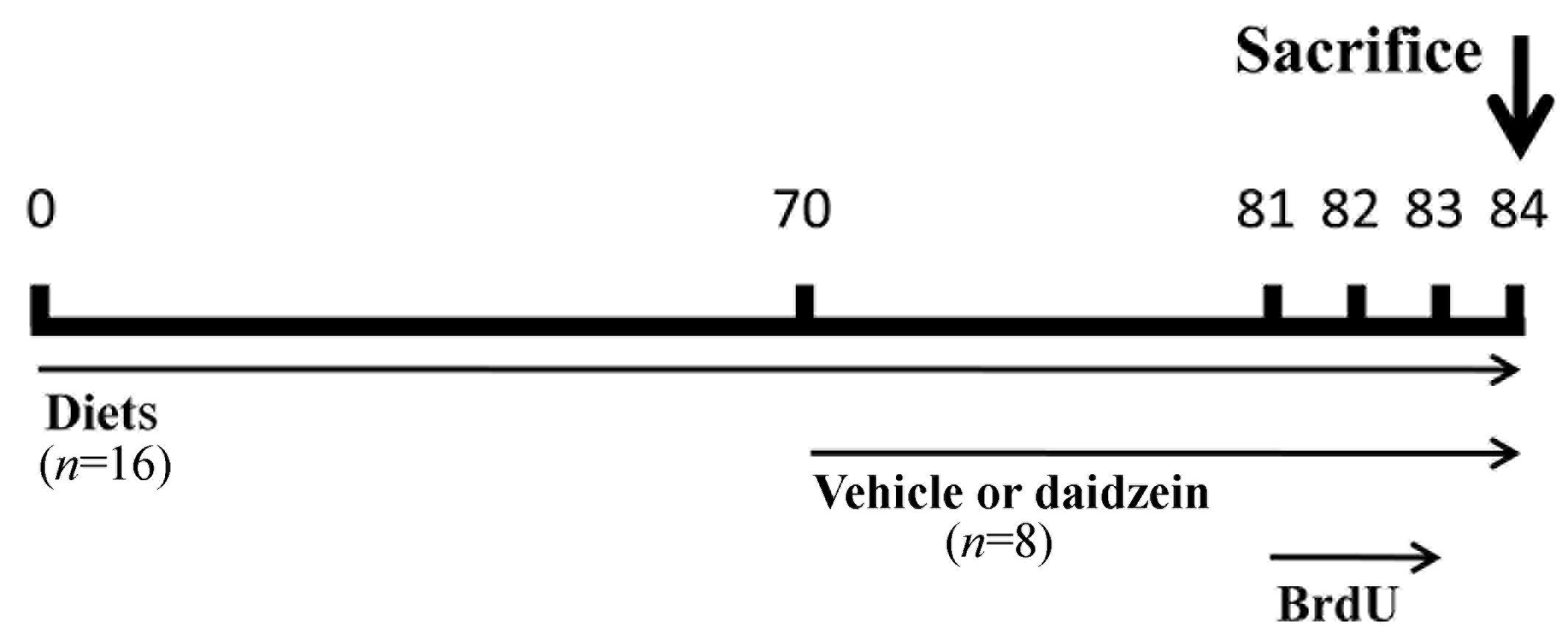

Supplement: Figure S1 — Experimental design used for feeding, daidzein administration and 5-bromo-2′-deoxyuridine (BrdU) injections. Animals were fed ad libitum for 84 days with two diets: a very high fat diet and a standard diet. After 70 days, when the weight curves achieved divergence and stabilization between both diets, rats received a daily intraperitoneal injection of daidzein (50 mg kg−1) or vehicle (Tocrisolve) for 13 days. Four days before sacrifice, animals received two daily intraperitoneal injection of BrdU (50 mg kg−1) at 10 hours intervals (8 a.m., 6 p.m.) for three consecutive days. Two hours after the last dose of daidzein, rats were sacrificed to collect blood samples and the brains. (TIF) [file pone.0064750.s001.tif]
