# Supplementary material for: Pharmacological Administration of the Isoflavone Daidzein Enhances Cell Proliferation and Reduces High Fat Diet-Induced Apoptosis and Gliosis in the Rat Hippocampus
Source: PLoS One. 2013 May 31;8(5):e64750. doi: 10.1371/journal.pone.0064750 (PMC3669353; doi:10.1371/journal.pone.0064750)
Supplement: Methodology S1 — Extensively description of methodology and antibodies used in the present study. (DOCX) [file pone.0064750.s002.docx]

**SUPPORTING METHODOLOGY**

*Ethics Statement*

The protocols for animal care and use were approved by the Ethic and Research Committee at the Hospital Carlos Haya and Universidad de Málaga. All experimental animal procedures were carried out in strict accordance with the European Communities directive 86⁄609⁄ECC (24 November 1986) and Spanish legislation (BOE 252⁄34367-91, 2005) regulating animal research. All efforts were made to minimize animal suffering and to reduce the number of animals used.

*Animals*

Male Wistar rats (approximately 250 g, 10–12 weeks old; Charles Rivers, Barcelona, Spain) were housed individually in cages maintained in standard conditions (Servicio de Estabulario, Facultad de Medicina, Universidad de Málaga) at 20 ± 2 ºC room temperature, 40 ± 5% relative humidity and a 12-h light ⁄ dark cycle with dawn and dusk effect.

*Food/caloric intake and body weight.*

Rats (*n*=16 per group) were fed *ad libitum* for 12 weeks with two different types of diets: a very high-fat diet (HFD, 60% fat diet; D12492, Brogaarden, Gentofke, Denmark) and a standard diet (STD, 10% fat diet-D1245B). The STD consisted of 3.85 kcal g^-1^ (20% protein, 70% carbohydrates and 10% fat) and the HFD had 5.24 kcal g^-1^ (20% protein, 20% carbohydrates and 60% fat). The accumulated food/caloric intake and the body weight gain were measured every 2-3 days for 12 weeks (Fig. S1). Treatment was administered when the body weight curves showed a clear divergence and stabilization between the two diets. This was accomplished after 10 weeks of diet.

*Subchronic daidzein treatment*

After 10 weeks, half of each group of diet-fed rats (*n*=8) received a daily intraperitoneal (i.p.) injection (09:00 a.m.) of, either 50 mg kg^-1^ daidzein (7[hyphen]β-glucoside; LC Laboratories, Worburn, USA) dissolved in 10% Tocrisolve (Tocris, Bristol, United Kingdom) and saline, or vehicle (1 ml kg^-1^ of 10% Tocrisolve in saline) over 13 days, while the diets remained unchanged (Fig. S1). The dose was selected on the basis of the acute effects of this isoflavone on a feeding test [4]. Cumulative food/caloric intake and body weight gain were measured every day over the 13 days of treatment. Thus, the following four experimental groups were generated: STD-vehicle, STD-daidzein, HFD-vehicle and HFD-daidzein.

*Glucose tolerance test*

At 30 minutes before starting the glucose tolerance test (GTT), 50 mg kg^-1^ daidzein or vehicle (1 ml kg^-1^ of 10% Tocrisolve in saline) was administered (i.p.) in 12-hour fasted rats fed STD and HFD. Twenty-five minutes later, tail blood samples were subsequently collected at basal level (0 minutes) and 5, 10, 15, 30, 60 and 120 minutes after a glucose overload (i.p.) at a dose of 2 g/kg body weight. Glucose levels were determined using a standard glucose oxidase method. GTT experiments were performed with groups of eight animals (*n*=8).

*BrdU administration*

5´-bromo-2´-deoxyuridine (BrdU, cat. no. B5002, Sigma, St. Louis, MO, USA) was dissolved at 15 mg ml^-1^ in 0.9% saline solution, and administrated (i.p.) at 50 mg kg^-1^ body weight twice per day at 10-hours intervals (08:00, 18:00 h), for 3 consecutive days (Fig. 1). The animals were killed 12 hours after the last injection of BrdU [20].

*Sample collection*

Previous to sacrifice, animals were anaesthetized (sodium pentobarbital, 50 mg kg^-1^ body weight, i.p.) two hours after the last dose of treatment in a room separate from the other experimental animals. Blood samples were briefly collected from the orbital cavity into tubes containing EDTA-2Na (1 mg/ml blood) and centrifuged (1600 *g* for 10 min, 4ºC), and all plasma samples were frozen at -80ºC for biochemical and hormonal analysis. Animals were then transcardially perfused with 4% paraformadehyde in 0.1 M phosphate buffer (PB) and brains were dissected and kept in the same fixative solution overnight at 4ºC. Brains were then cut into 40-μm-thick coronal sections, divided in five parallel series, using a vibratome (Leica VT1000S). Sections were stored at 4ºC in PB with 0.002% (w/v) sodium azide until further used for immunostaining.

*Biochemical and hormonal analysis*

Plasma metabolites and hormones were measured using commercial kits according to the manufacturer’s instructions. Glucose, triglycerides, total cholesterol and high-density lipoprotein (HDL)-cholesterol were analyzed in a Hitachi 737 Automatic Analyzer (Hitachi Ltd., Tokyo, Japan) in the Hematology Service at the Hospital Carlos Haya (Málaga, Spain). Plasma insulin, adiponectin and leptin levels were determined with three different enzyme-linked immunosorbent assay (ELISA) kits from Mercodia AB (Uppsala, Sweden), B-Bridge International, Inc. (Mountain View, CA, USA) and BioVendor (Modrice, Czech Republic), respectively. To perform the ELISA protocols in rat samples, we used 25, 100 and 100 μl of plasma to determine the insulin, adiponectin and leptin concentrations, respectively. Plasma testosterone and 17β-estradiol levels were determined using an Immulite®2000 immunoassay system (Siemens, Tarrytown, NY, USA). We used 200 μl of rat plasma to perform the immunoassays. In all cases, a calibration curve and an internal control were included in each assay. Cross-reaction with daidzein was not detected.

*General procedures for immunohistochemistry*

For the analysis of phospho-histone H3 and BrdU immunoreactivity in the SGZ of DG, and the immunohistochemical expression of total caspase-3, cleaved caspase 3, FosB, GFAP, Iba1 and ERα in the whole hippocampus, free-floating 30-μm-thick coronal sections from -2.12 to -4.16 mm Bregma level [21] were selected from one of eight parallel series obtained from each brain of the four experimental groups. The distance between sections was about 200 μm. Sections were first washed several times with PBS to remove sodium azide, and incubated in a solution of 3% hydrogen peroxide and 10% methanol in 0.1 M PB for 45 min at room temperature in darkness to inactivate endogenous peroxidase. After incubation in the primary and secondary antibody, immunolabelling was revealed with 0.05% diaminobenzidine (DAB; Sigma), 0.05% nickel ammonium sulfate and 0.03% H_2_O_2_ in PBS. After several washes in PBS, sections were mounted in slides treated with poly-l-lysine solution (Sigma), air-dried, dehydrated in ethanol, cleared with xylene and coverslipped with Eukitt mounting medium (Kindler GmBH & Co, Freiburg, Germany). Digital high resolution photomicrographs of the rat brain were taken under the same conditions of light and brightness ⁄contrast by an Olympus BX41 microscope equipped with an Olympus DP70 digital camera (Olympus Europa GmbH, Hamburg, Germany). Representative digital images were mounted and labelled using Microsoft PowerPoint 2007.

*BrdU immunohistochemistry*

Antibodies used were as follows [20]:

1. Mouse anti-BrdU is an IgG monoclonal antibody obtained from ascites by using BrdU-bovine serum albumin (72–73 kDa) as immunogen. Dilution: 1:2000. Source: Hybridoma Bank, Iowa City, IA, USA; ref. G3G4.
2. Biotinylated goat anti-mouse IgG (H+L). Dilution: 1:1000. Source: Pierce, Rockford, IL, USA; cat. no. 31800.

One immunostaining batch containing 8 sections per animal (*n*=8, four groups) was stained simultaneously to avoid variations in the intensity of staining due to the procedures. After inactivation of endogenous peroxidase and three washes in PBS for 10 min, DNA was denatured by exposing sections to 2 M HCl for 15 min at 37ºC, followed by two washes in 0.15 M borate buffer to neutralize pH. After three washes in PBS for 10 min, sections were incubated in a blocking solution containing 0.3% albumin, 0.3% triton X-100 and 0.05% sodium azide in PBS for 45 min. Sections were then incubated overnight in diluted primary antibody (mouse anti-BrdU) at 4ºC. The following day, sections were washed three times with PBS, incubated in secondary antibody (biotinylated goat anti-mouse) for 90 min, washed again in PBS, and incubated in avidin-biotin peroxidase complex (Pierce) diluted 1:250 in PBT in darkness at room temperature for 45 min. Finally, immunolabelling was revealed as was described above.

*Double immunofluorescence*

The following antibodies were used: rat anti-BrdU (1:2000; Accurate Chemical & Scientific, Westbury, NY, USA) and mouse anti-β3 tubulin (1:5000; Promega, Madison, WI, USA). Sections were pretreated as described above and incubated overnight at 4ºC with a cocktail of the primary antibodies. After washing in PB, sections were incubated for 2 h at room temperature with the secondary antibodies. The rat anti- BrdU was detected with goat anti-rat IgG labelled with Alexa 488 (1:1000; Molecular Probes, Invitrogen, Paisley, UK). The antibody β3-tubulin was detected with donkey anti-mouse IgG Alexa 594 (1:1000; Molecular Probes). The sections labeled by double immunofluorescence were visualized with a confocal microscope (Leica TCS NT; Leica Microsystems).

*Phospho-H3, caspase-3, FosB, GFAP, Iba 1 and ERα immunohistochemistry*

Specificity of primary antibodies used in this study was tested in previous studies and they were used as follow:

1. Rabbit anti-phospho-histone H3 (Ser10) is a purified IgG polyclonal antibody produced by immunizing rabbits with a KLH-conjugated peptide corresponding to amino acids 7-20 of human serine-10 phosphorylated histone H3. Dilution: 2 μg/ml. Source: Upstate, Lake Placid, NY, USA; cat. no. 06-570.
2. Rabbit anti-caspase-3 is an affinity purified IgG polyclonal antibody produced by immunizing rabbits with a synthetic peptide (KLH-coupled) corresponding to residues surrounding the cleavage site of human full length (35 kDa) and cleaved caspase-3 (17 kDa). Dilution: 1:200. Source: Cell Signaling Technology, Danvers, MA, USA; cat. no. 9662.
3. Rabbit anti-cleaved caspase-3 is a purified IgG polyclonal antibody produced by immunizing rabbits with a synthetic peptide corresponding to amino-terminal residues adjacent to Asp175 in human caspase-3 (17 kDa). Dilution: 1:500. Source: Cell Signaling; cat. no. 9661.
4. Rabbit anti-FosB is an affinity purified IgG polyclonal antibody raised against a peptide mapping within an internal region of mouse FosB (43 kDa). Dilution: 1:2500. Source: Santa Cruz Biotechnology, Santa Cruz, CA, USA; cat. no. sc-48.
5. Mouse anti-glial fibrillaric acidic protein (GFAP) is an affinity purified IgG monoclonal antibody from ascites of clone GA5 using purified GFAP (50 kDa) from pig spinal cord as immunogen. Dilution: 1:500. Source: Sigma; cat. no. G3893.
6. Rabbit anti-Iba-1 is a purified IgG polyclonal antibody produced by the affinity chromatography from rabbit antisera and raised against synthetic peptide corresponding to C-terminus of Iba1. Dilution: 1:1000. Source: Wako, Osaka, Japan; cat. no. 019-19741.
7. Rabbit anti-estrogen receptor alpha (ERα) is an affinity purified IgG polyclonal antibody raised against amino acids 2-185 of human ERα (66 kDa). Dilution: 1:200. Source: Santa Cruz Biotechnology; cat. no. sc-7207.

The secondary antibodies used were as follows.

1. Biotinylated goat anti-mouse IgG. Dilution: 1:500. Source: Sigma; cat. no. B7264.
2. Biotinylated donkey anti-rabbit immunoglobulin. Dilution: 1:500. Source: Amersham, Little Chalfont, England; cat. no. RPN 1004.

One immunostaining batch containing 5 sections per animals (*n*=8, four groups) was stained simultaneously to avoid variations in the intensity of staining due to the procedures. Sections were incubated in sodium citrate (50 mM in dH2O, pH 6) for 30 min at 80°C, followed by three washes in PBS for 10 minutes each time, previous inactivation of the endogenous peroxidase. Sections were incubated in blocking solution containing 10% serum (donkey or goat), 0.3% triton X-100, and 0.1% sodium azide in PBS for 1 hour. Then, sections were incubated overnight in diluted primary antibody at 4ºC. The following day sections were washed three times with PBS, incubated in a secondary antibody for 1 hour, washed again in PBS, and incubated in ExtrAvidin peroxidase (Sigma, St. Louis, MO) diluted 1:2000 in darkness at room temperature for 1 hour. Finally, immunolabelling was revealed as was described above.

*Quantification of phospho-H3, caspase-3, FosB, GFAP and Iba1-immunoreactive cells*

Phospho-H3, caspase-3, FosB, GFAP, Iba1-immunoreactive (-ir) nuclei or cells that came into focus were manually counted using a standard optical microscope with the 40× objective (Nikon Instruments Europe B.V., Amstelveen, The Netherlands) coupled to the NIS-Elements Imaging Software 3.00 (Nikon). Phospho-H3 and BrdU-ir nuclei in the SGZ of dentate gyrus, and caspase-3, FosB, GFAP and Iba1-ir cells in the three hippocampal areas (DG, CA3 and CA1) were counted from -2.12 to -4.16 mm Bregma levels for each animal (*n*=8). Nuclei and cell counts were performed in a single cerebral hemisphere in all cases. For GFAP immunostaining, a representative counting frame (40×) was evaluated in each area (DG, CA1 and CA3) and section analyzed. GFAP-ir cells located in the uppermost side that came into focus while moving down through the thickness of the section were counted. GFAP-positive fibers without a visible body cell were ignored. Overall, quantification was expressed as the total number of BrdU-ir cells or the average number of caspase-3, FosB, GFAP and Iba1-ir cells per area (mm^2^) for each experimental group.

*Quantification of GFAP and ERα immunoreactivity*

Digital high-resolution microphotographs of the hippocampus were taken with a 10× objective under the same conditions of light and brightness/contrast with an Olympus BX41 microscope equipped with an Olympus DP70 digital camera. Densitometric quantification of the immunoreactivity of representative areas was determined using the analysis software ImageJ 1.38X (NIH, USA). On each tissue section, we focused on CA1 and CA3 areas of Ammom’s horn and DG. For both CA areas, we considered the following layers: stratum oriens (SO), stratum pyramidale (SP), stratum radiatum (SR), stratum lucidum (SL) and stratum lacunosum-moleculare (SL-M). For DG, we considered following layers; the molecular layer (ml), the granular cell layer (gcl) and the polymorphic cell layer (pcl).

*Statistical analysis*

Data are represented as mean ± s.e.m. from at least six animals. Kolmogorov-Smirnov normality tests indicated that all data followed a Gaussian distribution (*P*>0.1), so we selected a parametric statistical test. Statistical analysis was performed using two-way ANOVA with the two factors being diet (STD and HFD) and treatment (vehicle and daidzein), followed by Bonferroni *post hoc* test for multiple comparisons. P<0.05 was considered to be significant.
